# The role of hopelessness in mediating the relationship between income loss and delaying and foregoing healthcare: Evidence from repeated cross-sectional waves of the Household Pulse Survey

**DOI:** 10.1371/journal.pmen.0000395

**Published:** 2025-07-31

**Authors:** Christopher R. Gustafson, Kathleen R. Brooks, Syed Imran Ali Meerza, Amalia Yiannaka, Eliana Zeballos

**Affiliations:** 1 Department of Agricultural Economics, University of Nebraska-Lincoln, Lincoln, Nebraska, United States of America; 2 Department of Agriculture, Arkansas Tech University, Russellville, Arkansas, United States of America; 3 U.S. Department of Agriculture, Economic Research Service, Southwest Washington, Washington, D.C., United States of America; UCL: University College London, UNITED KINGDOM OF GREAT BRITAIN AND NORTHERN IRELAND

## Abstract

Research has documented direct negative impacts of crises, such as COVID-19, on people’s mental health. However, evidence is limited about how these events impact decision-making through direct influences on choices, or by indirectly changing decision-making through mental health effects. Research on avoidance behaviors suggests that affective states influence decisions to access healthcare and receive diagnoses. While there is significant evidence that hopelessness related to a potential health threat impacts decisions to learn about that threat, affective responses to crises may also cause spillovers to decision-making in other domains. In this study, we examine linkages between exposure to a stressor (COVID-19-related income loss), feelings of hopelessness, and foregoing or delaying healthcare across multiple cross-sections of the US Census’s Household Pulse Survey, featuring 2.76 million survey responses collected between April 23, 2020, and July 5, 2021. After removing observations with missing data for dependent variables, the final sample size is just under 2.3 million responses. We conduct ordered logistic regressions of the relationship of income loss with hopelessness levels, and logistic regression of the relationship of income loss and hopelessness levels on health care access. We additionally report versions of the regressions with demographic variables and time and state fixed effects to control for important factors related to those variables. We conduct a mediation analysis to estimate the pathway of income loss acting through hopelessness. The analyses find that experienced income loss predicts significantly higher levels of hopelessness (odds ratio (OR)=1.68 (95%CI = 1.67, 1.69)). Both hopelessness and income loss are, in turn, associated with healthcare access—an increased likelihood of foregoing and/or delaying needed medical care (e.g., hopelessness nearly every day (OR=4.18, 95%CI = 4.13, 4.23), experienced income loss OR=1.25, 95%CI = 1.24, 1.26)). A mediation analysis confirms that hopelessness significantly and consistently mediates approximately 30% of the relationship of COVID-19 income loss to foregoing/delaying healthcare.

## 1. Introduction

A large body of research examines the influence of affect—emotions, moods, and feelings—on decision-making [1,2]. For instance, negative affective states, such as sadness, have been found to increase people’s perceived probability of an adverse event occurring [3,4], leading them to be more risk averse during decision-making [5]. Hopelessness, characterized by feelings of powerlessness to effectively attain desired outcomes or respond to challenges, emerges as a significant predictor of avoidance behaviors in critical health domains [6–9]. This pattern of avoidance has been observed in various settings, including areas such as nutrition information [10]; disease testing (e.g., cancer, diabetes, HIV) [11–16], and critical public health topics like antimicrobial resistance [9].

These studies of avoidance behaviors all represent situations in which individuals anticipate negative affect if unfavorable information—such as about a disease state—is received. While this has been a common conceptualization of the role of affect in decision-making, affect can influence the decision-making process through multiple routes [17]. For instance, exogenous forces can impact emotions and influence unrelated decision processes. Research illustrating this case includes findings that day-to-day variation in weather conditions influences investment decisions, stock prices, and purchases of consumer goods [18–21], or that individuals experiencing higher levels of hopelessness after exposure to COVID-19-related income loss are more likely to avoid information about an unrelated health threat [22].

In other cases, exogenous forces may both impact emotions but also be expected to directly influence the decision itself. A potential example of this case is income loss, affective response to income loss, and decisions to access healthcare. Income loss may decrease an individual’s ability to pay for healthcare, while affective response to income loss may have a further impact on the decision process. There is little evidence about affect-mediated spillovers from one domain to another, but it has important implications for the breadth of impact that events that engender affective responses have.

A well-documented impact of COVID-19 was a marked increase in negative affect, such as feelings of hopelessness [23,24] and depressive symptoms [25]. A significant increase in the delay or foregoing of medical care was documented during the early stages of the COVID-19 pandemic [26], which was estimated to have real consequences—a “crisis of undiagnosed cancers” was reported [27], with estimates that thousands of additional deaths would result from colorectal and breast cancers [28] and other diseases [29]. Individuals were more likely to delay non-critical care, such as dental appointments or regular checkups, than to forego critical care, such as surgical procedures [30]. Both actions pose significant threats to human health, mirroring the urgency of COVID-19, and were therefore likely to be highly salient to respondents. However, affect—including hopelessness—can influence these decisions. The COVID-19 pandemic undoubtedly heightened public awareness of health concerns, making individuals more likely to think about these issues [31] and thus potentially less likely to avoid care [32]. However, while COVID-19 and non-COVID health problems represent significant threats to health, they often involve distinct risk factors, transmission routes, and treatment options. Therefore, accessing treatment and preventative care for non-COVID health problems was crucial.

In this study, we conduct a pre-registered analysis of 32 weeks of survey data from repeated cross-sections of the U.S. Census’s Household Pulse Survey to examine whether higher levels of hopelessness associated with COVID-related income loss are associated with a greater likelihood of foregoing or delaying needed medical care. We use mediation analysis to estimate the proportion of the variation in decisions to access care that are explained directly by income loss and indirectly through feelings of hopelessness. We estimate two versions of the models: one that only includes the variables of interest described above; the second includes demographic variables, including the respondent’s sex, education, age, race/ethnicity, household income, health insurance status, as well as time and state of residence fixed effects to control for unobservable characteristics such as differences in state policies that might affect the variables of interest. The analyses find that hopelessness consistently and significantly mediates the relationship between income loss and foregoing or delaying healthcare despite large changes in experienced and expected income loss throughout the study period.

## 2. Materials and methods

### 2.1. Ethics statement

A review of the research protocol by the University of Nebraska-Lincoln Institution Review Board declared that the research conducted in this paper is not human subjects research due to the sole use of publicly available, de-identified secondary data. The IRB’s review concluded that, “we have determined that this project does not meet the definition of human subjects research at 45 CFR 46. Furthermore, this project does not require IRB approval. This project does not meet the definition of human subjects research because it does not seek to obtain information about an individual either through intervention/interaction or through privately identifiable information, as all information will be obtained through publicly available datasets (Household Pulse Survey) through federal sources (US Census Bureau). Based on this assessment, the project will be identified as not human subjects research.”

### 2.2. Survey design and data

This study uses de-identified data downloaded from the freely available public use files from 32 weeks of the repeated cross-sections of the US Census Bureau’s Household Pulse Survey (HPS). The US Census Bureau’s HPS, a survey conducted in a two weeks on/two weeks off collection approach starting April 23, 2020, collected data on the decision to forego or delay needed medical treatment in the previous four weeks, feelings of hopelessness, income loss, and demographic characteristics, as well as the state the respondent resided in, and other variables. We excluded data after wave 33 due to changes in the questions asked, which took effect in wave 34.

We pre-registered all analyses reported in this paper, which covers HPS data from weeks 1–20 and 22–33. We excluded HPS week 21 data because data on hopelessness from that survey week were previously examined and compared with hopelessness rates collected in a custom survey on information avoidance during COVID-19 [22]. The pre-registration is available at https://osf.io/d67y8.

**Fig 1:**
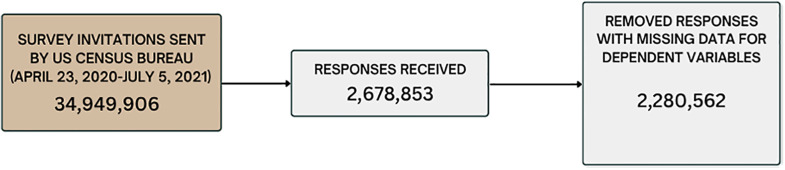
Flow diagram of sample recruitment, responses, and data cleaning.

We downloaded public-use data for weeks 1–20 and 22–33 of the HPS to examine healthcare access outcomes (available at: https://www.census.gov/programs-surveys/household-pulse-survey/data/datasets.2020.html#list-tab-1264157801). Fig 1 presents a flow diagram of sample recruitment, responses, and data cleaning. Across these 32 weeks, the Census Bureau sent invitations to 34,949,906 individuals, receiving 2,678,853 responses (7.7% response rate). Because respondents were not required to answer all questions, some surveys did not provide data for the healthcare access or hopelessness questions; data from these surveys were omitted from the analysis. We present the distribution of demographic characteristics by whether the respondent provided an answer to questions about hopelessness and healthcare access in S1 Table. Individuals not responding to these questions had less education and income (though most declined to report income), were more likely to be Hispanic/Latino, non-white, and female (though only by 0.5 percentage points in the case of female), and were slightly younger than those who responded to the questions. Many of the characteristics with higher non-response rates, such as low education, income, and minority status, were associated with poorer mental health outcomes during COVID [33–35]. We obtained usable data from 2,280,562 responses for the pre-registration of weeks 1–20 and 22–33 [36]. We provide week-by-week sample invitation and response rates as supplementary materials (S2 Table).

We use data from the HPS to examine the effect of COVID-related stressors on decisions to forego or delay needed medical treatment, examining both direct and indirect—through hopelessness—pathways. The survey included two relevant healthcare questions. The first question asked whether the respondent had needed medical care in the previous four weeks that was not caused by a COVID-19 infection but had not received it. The second question asked whether the respondent had delayed getting medical care in the previous four weeks. The potential responses to both questions were “Yes” or “No,” though respondents were able to move past the question without selecting either answer. We omit data from individuals who did not provide an answer to these two questions.

The set of independent and mediator variables retrieved from the HPS datasets were hopelessness, income loss, and demographic variables collecting information about the respondent’s gender, age, education, race/ethnicity, and household income, whether the members of the household had health insurance, as well as the survey wave and the state in which the individual resided. We examine income loss because it has been identified as a key contributor to decreases in mental health [37]. There were two income-loss questions in the HPS surveys. The first asked if “you or anyone in your household experienced a loss of employment income since March 13, 2020?” for survey waves 1–27; starting with wave 28, the question asked, “Have you, or has anyone in your household experienced a loss of employment income in the last 4 weeks?” We use this variation in the temporal proximity of the income loss event to identify differences in impact on feelings of hopelessness and healthcare decisions. This also separates the data into time periods in which greater (waves 1–27) or fewer (waves 28–33) restrictions on schools, businesses, and individuals were in place, which may have affected access to or time for (e.g., if children were at home instead of at school) obtaining healthcare. In the latter period, this serves to isolate the impact of hopelessness and income loss on healthcare access.

The second income loss-related question focused on expected losses. This question asked, “Do you expect that you or anyone in your household will experience a loss of employment income in the next 4 weeks because of the coronavirus pandemic?” This question was consistent throughout the 32 waves of the survey we examined. For both experienced and expected income loss questions, the available responses were “yes” and “no,” though individuals could again proceed without responding; those who did not respond to the income loss questions were categorized as “Did not respond” for the analysis.

Hopelessness data were collected in terms of the number of days in the previous seven days that the individual reported feeling hopeless. The survey provided categorical options for responses to this question: “nearly every day,” “more than half the days,” “several days,” or “not at all.” As with the healthcare access question, some individuals did not respond to this question. Data from surveys that did not contain a response to the hopelessness question were not included in the regression analyses, although we do report all data (including those who did not respond) in Table 2.

### 2.3. Analyses

We assess the relationship between the COVID-19-related income loss variables, variation in feelings of hopelessness, and decisions to forego and/or delay medical care. In our first analysis, we examine the relationship of participants’ experienced or expected income loss during the COVID-19 pandemic to feelings of hopelessness. We conduct this analysis using an ordered logistic regression model. We report two versions of the regression results: one with experienced/expected income loss as the only independent variable, and a second version that also includes common demographic characteristics, such as gender, age, education, race/ethnicity and household income, whether the household had health insurance, as well as week and state fixed effects. The second regression is important as policies enacted in response to COVID-19 varied from state to state, which influenced the availability of healthcare in certain cases, and also affected mental health outcomes [38].

In our second analysis, we examine the relationship between hopelessness and foregoing or delaying needed healthcare using binary logistic regression models. The outcome variable for foregoing/delaying needed healthcare was the respondent’s report that they had foregone or delayed needed healthcare in the previous four weeks. We include income loss in addition to hopelessness to identify whether this COVID-19-related stressor directly influenced healthcare access decisions. In a second version of the regression, we additionally included demographic variables, health insurance status, and time and state-specific fixed effects to control for differences in policies and other unobserved state and time-related variables that could affect healthcare access.

We conducted these analyses on the full sample (weeks 1–33, omitting 21), as well as the two sub-samples distinguished by the form of the experienced income loss question (weeks 1–27 (omitting 21) vs. weeks 28–33), both with and without demographic and other state and temporal controls. We additionally compared estimates with and without sampling weights to check for bias but ultimately report results without sampling weights. First, the constructs under investigation—hopelessness and healthcare access decisions—do not have a clearly defined or theoretically justified relationship with the HPS sampling frame. As such, the use of weights would not correct for any known bias related to the sampling design in this context. Second, unnecessarily applying survey weights in regression models when the model does not align with the survey sampling design can increase variance and reduce statistical efficiency, particularly when the weights are highly variable or when the model does not align with the survey design [39]. For key models we calculate and report average marginal effects [40].

Finally, to estimate the role of hopelessness in mediating the impact of COVID-related stressors on decision-making, we conducted a mediation analysis that estimates the impact of income loss in response to COVID-19 on both delaying and foregoing medical care through feelings of hopelessness. Mediation analysis uses bootstrapping techniques to establish the statistical significance of estimated relationships of the independent and mediator variables with the dependent variable [41–45]. For the mediation analyses, we convert the income loss variable from a multi-outcome categorical variable—experienced income loss, expected income loss, neither experienced nor expected income loss—to a binary variable (experienced or expected income loss = 1; neither experienced nor expected income loss = 0) as required by the R ‘mediation’ package [46] used for the analysis. For all analyses, we interpret results with p-values < 0.05 to be statistically significant.

## 3. Results

**Fig 2:**
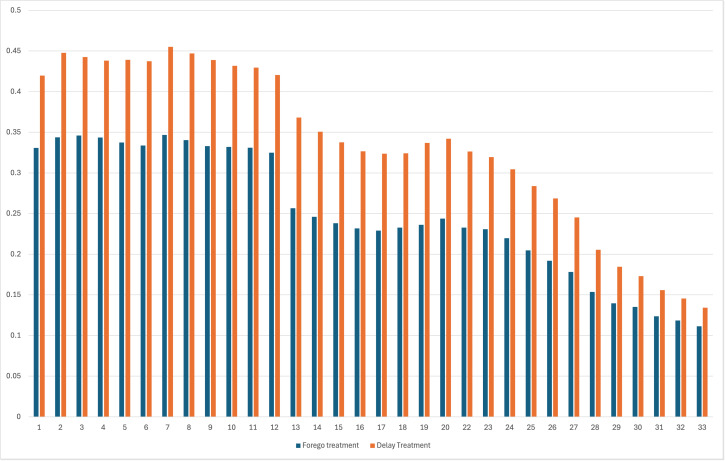
Proportion of participants foregoing or delaying treatment in previous four weeks by survey wave (April 2020–July 2021).

First, we report summary statistics about foregoing or delaying needed medical care. Approximately 25 percent and 35 percent of the HPS respondents reported foregoing or delaying needed medical treatment, respectively, in the previous four weeks across the study period (April 2020-July 2021). However, there were significant changes in percentages of respondents reporting foregoing or delaying medical care across waves of the survey (see Fig 2). The percentage of respondents who delayed medical care decreased from highs of over 45 percent to 13 percent at the end of the study period. Respondents reporting foregoing medical care peaked at nearly 35 percent in the early part of the study period and declined to 11 percent at the end of the study period.

Table 1 summarizes the demographic characteristics of participants in the HPS survey, compared to the overall U.S. population. The HPS sample is slightly more female, older, more educated, and has higher median household income than the overall adult U.S. population.

**Table 1:** Summary statistics.

	HPS Survey Waves 1–20, 22–33	U.S. Population
Female (%)	59%	51%
Age (% Adults < 65)	74%	79%
Education (% ≥ bachelor’s degree)	55%	32%
Household income (Median)	$87,500	$62,843

HPS = Household Pulse Survey.

Notes: Data are from 2,678,853 responses to Phases 1-3.1 (Weeks 1–20 and 22–33) of the Census HPS (Apr. 23, 2020-Jul. 5, 2021); US population data from US Census Bureau Quick Facts [47].

**Fig 3:**
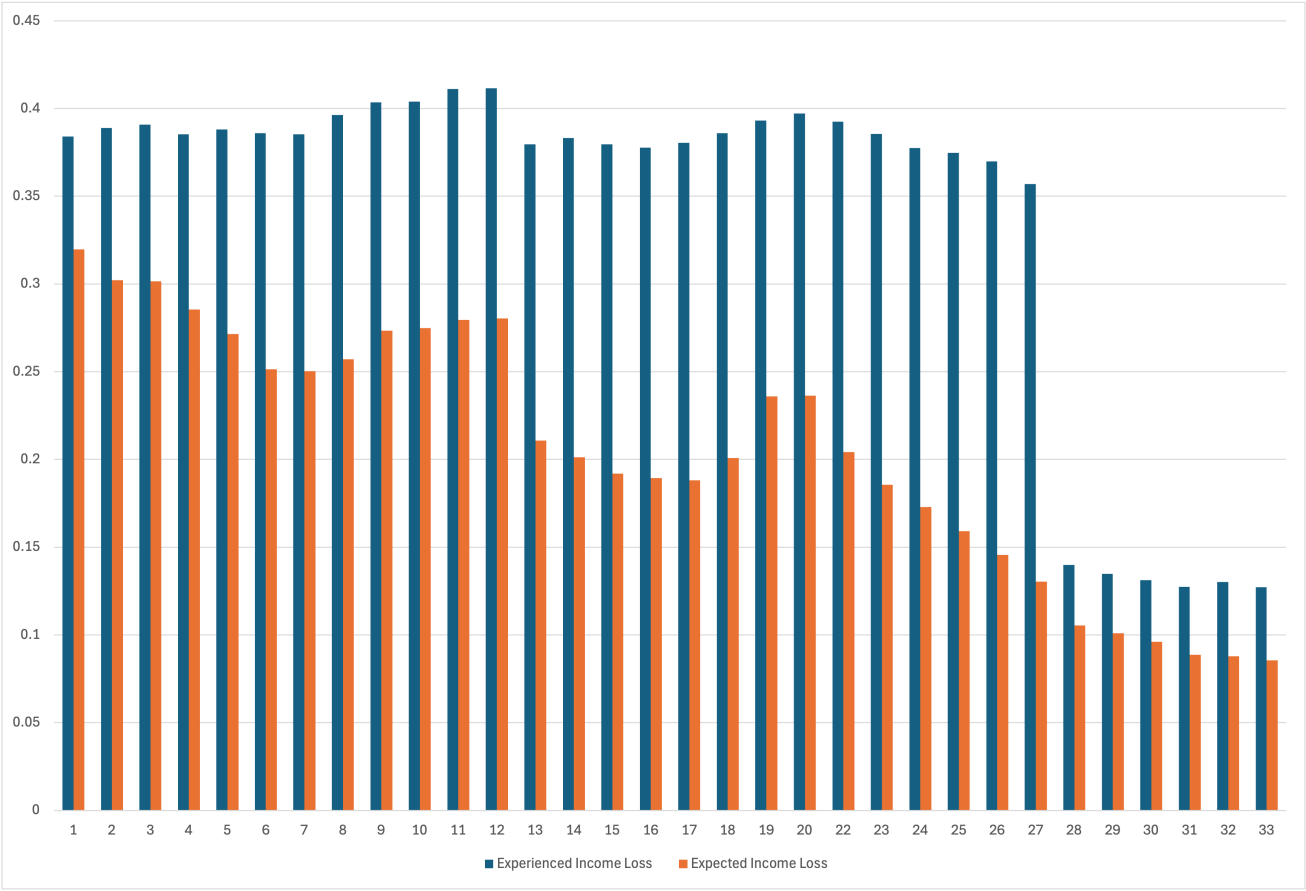
Proportion of respondents who experienced income loss (defined as “from the start of the pandemic” for waves 1–27 versus “during the past four weeks” for waves 28–33) or expecting income loss during the study period.

Next, we examine income loss variables. Fig 3 presents mean responses to questions about experienced and expected income loss. Recall that experienced income loss was initially asked as any income loss occurring from the onset of the pandemic (waves 1–27) and then changed to income loss experienced during the previous four weeks (waves 28–33). On average, around 40 percent of respondents reported experiencing income loss from the beginning of the COVID-19 pandemic to the date they answered the survey (question format during waves 1–27), while an average of 13 percent reported having experienced income loss during the previous four weeks during waves 28–33. Across the survey period, 21 percent of respondents expected to incur a loss of income within the next four weeks, though this decreased over the course of the study period from a high of 32 percent during wave 1 to a low of 8.5 percent in wave 33.

We next compare levels of hopelessness between the HPS data (waves 1–33, omitting wave 21) and a pre-pandemic 2019 CDC National Health Interview Survey [48,49], which is reported in Table 2.

**Table 2:** Distribution of responses to question about feelings of hopelessness in the previous seven days.

	Not at all	Several days	More than half the time	Nearly every day	Prefer not to answer
HPS1-3.1 (N = 2,678,853)	45.5%	26.3%	7.5%	7.5%	13.2%
NHIS (N = 31,289)	82.1%	12.5%	2.2%	2.8%	0.3%

Notes: Data from US Census Household Pulse Survey, Phases 1-3.1 (Apr. 23, 2020-Jul. 5, 2021); and 2019 National Health Interview Survey [48,49].

**Fig 4:**
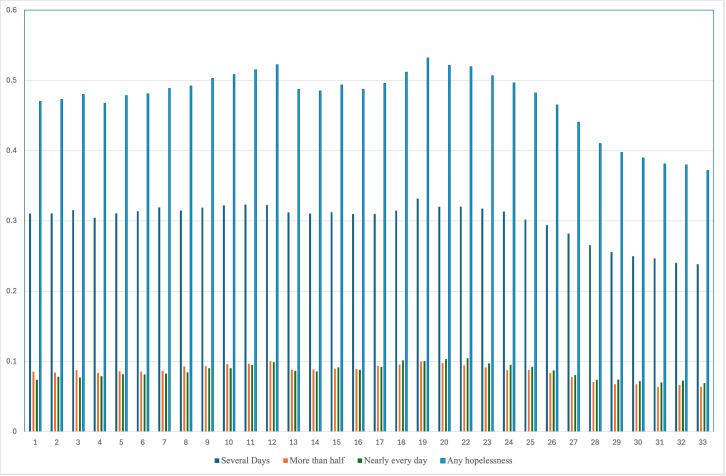
Proportion of respondents reporting different levels of hopelessness during the previous week by survey wave.

Respondents to the pandemic-era HPS surveys exhibited markedly higher levels of hopelessness than respondents to the 2019 NHIS survey. Overall, 41.3 percent of HPS respondents reported feeling some level of hopelessness during the previous seven days. In contrast, the pre-pandemic NHIS survey demonstrated significantly lower levels of hopelessness during the previous week, with 17.6 percent of respondents reporting hopelessness across all categories. Hopelessness decreased during the HPS data from a high of 53 percent in survey wave 19 to a low of 37 percent in wave 33. Fig 4 presents the proportion of respondents reporting any level of hopelessness during the previous seven days. Other studies documented worsening mental health during the pandemic [50], with effects on depressive and mood disorder symptoms lasting longer than other mental health conditions [51]. While many of these studies concluded data collection in mid-2020 [52], the data in Fig 4 display multiple peaks in feelings of hopelessness.

### 3.1. The impact of income loss on hopelessness

To estimate the impact of income loss on hopelessness, we analyzed 32 waves of HPS data. The ordinal logistic regression model analyzing the association between experienced or expected income loss and the frequency of feeling hopeless over the previous seven days, with and without demographic controls, health insurance, and state- and time-specific fixed effects, is reported in Table 3. The findings indicate that income loss—whether experienced or expected—predicts a significantly greater likelihood of feeling hopeless. Experienced income loss increases the odds of higher levels of hopelessness by approximately 1.4 times when controlling for demographic, insurance, state, and time variables (and 1.7 times without controls). Expecting an income loss within the next four weeks has an even stronger relationship with the likelihood of feeling hopeless. Expecting income loss increases the odds of experiencing higher levels of hopelessness by 1.7 to 1.8 times (with and without the inclusion of control variables). The temporal specificity of the income loss questions appears to be important. When the experienced income loss question changed to ask specifically about the previous four weeks in wave 28 (rather than any time since the beginning of the pandemic in waves 1–27), the odds of experienced income loss predicting higher levels of hopelessness increased to nearly 1.8 (with controls) and 2.2 without controls, which is equivalent to the magnitude of estimates on the expected income loss variables, which always referred to a four-week period. We include the full results of the regression, including demographic, health insurance, and spatiotemporal control variables, in S3 Table. Being older, more educated, having higher household income, and having health insurance all had a significant protective effect relative to feelings of hopelessness. Being female, however, predicted increased odds of hopelessness.

**Table 3:** Adjusted Odds ratios and 95% confidence intervals of the impact of the experienced or expected loss of income on experienced hopelessness during the previous seven days from ordered logistic regression analysis of HPS Survey data with and without demographic and state/time control variables.

	Waves 1–33 aOR (95% CI)	Waves 1–27 aOR (95% CI)	Waves 28–33 aOR (95% CI)	Waves 1–33 aOR (95% CI)	Waves 1–27 aOR (95% CI)	Waves 28–33 aOR (95% CI)
INCOME LOSS						
Experienced	1.68 (1.67, 1.69)	1.61 (1.60, 1.62)	2.18 (2.12, 2.23)	1.39 (1.38, 1.40)	1.37 (1.36, 1.38)	1.77 (1.73, 1.82)
Experienced – Did not respond	1.09 (1.02, 1.17)	1.11 (1.03, 1.20)	1.08 (0.93, 1.24)	1.12 (1.04, 1.21)	1.10 (1.01, 1.20)	1.15 (0.98, 1.36)
Expected	1.83 (1.81, 1.84)	1.81 (1.79, 1.82)	1.96 (1.90, 2.02)	1.73 (1.72, 1.75)	1.72 (1.71, 1.74)	1.82 (1.76, 1.87)
Expected – Did not respond	1.30 (1.24, 1.37)	1.33 (1.26, 1.40)	1.15 (1.00, 1.31)	1.32 (1.24, 1.40)	1.32 (1.24, 1.41)	1.20 (1.03, 1.40)
Demographic and Spatiotemporal Controls	No	No	No	Yes	Yes	Yes

Notes: Data from US Census Bureau Household Pulse Survey, Weeks 1–20 and 22–33.

**Fig 5:**
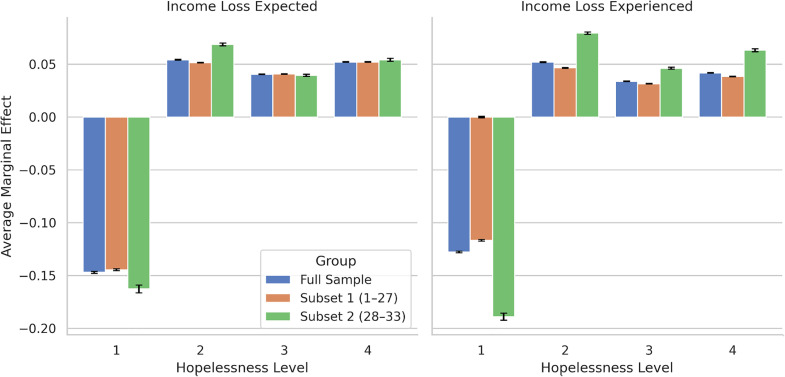
Average marginal effects of expected income loss and experienced income loss on each hopelessness level experienced during the previous seven days (1 = not at all; 2 = several days; 3 = more than half the time; 4 = nearly all the time).

Fig 5 presents average marginal effects for experienced income loss and expected income loss (relative to no experienced/expected income loss) for each level of hopelessness. Individuals who expected or experienced income loss had significantly lower probabilities (ranging from 11.7 to 18.9 percentage points) of experiencing hopelessness level 1, which represents experiencing hopelessness “not at all,” over the previous seven days. On the other hand, expected or experienced income loss significantly increased the probability of experiencing hopelessness on several days (2), more than half the time (3), or nearly all the time (4). These estimated impacts ranged from 4.7 to 7.9 percentage points for “several days,” 3.1 to 4.6 percentage points for “more than half the time,” and 3.9 to 6.3 percentage points for “nearly every day.” The size of the range is in every case due to the change in definition of the experienced income loss variable, which changed in week 28 from asking about experiencing income loss any time since the onset of the pandemic (weeks 1–27) to experiencing income loss during the past four weeks (weeks 28–33). The closer temporal proximity of the income loss experience leads to larger estimates of the impact of that loss on hopelessness in weeks 28–33 than in weeks 1–27.

### 3.2. The relationship of hopelessness to healthcare access

Next, we examine whether general feelings of hopelessness significantly predict foregoing or delaying needed medical care (Table 4–6). To assess the robustness of this relationship, we estimated four versions of a logistic model that includes the hopelessness variable, an economic COVID-19 stressor—experienced/expected income loss—as well as demographic characteristics, health insurance status, and state and week fixed effects. The analysis reveals a strong and consistent association between higher feelings of hopelessness and increased likelihood of foregoing or delaying medical care.

**Table 4:** Odds ratios and 95% confidence intervals of the association of feeling hopeless to foregoing non-COVID-related needed medical care in previous four weeks using data from HPS waves 1–20 and 22–33 with and without demographic, health insurance, and state/time control variables.

	Forego Needed Medical Care					
	(I) aOR (95% CI)	(II) aOR (95% CI)	(III) aOR (95% CI)	(IV) aOR (95% CI)	(V) aOR (95% CI)	(VI) aOR (95% CI)
Intercept	0.21 (0.20, 0.21)	0.26 (0.26, 0.27)	0.18 (0.18, 0.18)	0.23 (0.22, 0.24)	0.36 (0.35, 0.37)	0.15 (0.15, 0.16)
HOPELESS						
Nearly every day	4.67 (4.62, 4.72)	–	3.93 (3.89, 3.97)	4.64 (4.59, 4.70)	–	4.18 (4.13, 4.22)
More than half the days	3.34 (3.30, 3.37)	–	2.92 (2.89, 2.95)	3.21 (3.17, 3.25)	–	2.96 (2.92, 2.99)
Several days	2.17 (2.16, 2.19)	–	2.00 (1.99, 2.01)	2.09 (2.08, 2.11)	–	1.99 (1.97, 2.00)
INCOME LOSS						
Experienced	–	1.42 (1.41, 1.43)	1.26 (1.25, 1.27)	–	1.33 (1.32, 1.34)	1.25 (1.23, 1.26)
Experienced-No Response	–	1.10 (1.01, 1.19)	1.08 (0.99, 1.17)	–	1.13 (1.03, 1.24)	1.11 (1.01, 1.22)
Expected	–	1.89 (1.87, 1.90)	1.65 (1.64, 1.67)	–	1.69 (1.67, 1.71)	1.50 (1.48, 1.51)
Expected-No Response	–	1.44 (1.36, 1.53)	1.37 (1.28, 1.46)	–	1.40 (1.30, 1.50)	1.32 (1.23, 1.41)
Demographic and Spatiotemporal controls	No	No	No	Yes	Yes	Yes
Akaike Information Criterion	2,485,802	2,546,589	2,450,446	2,025,596	2,083,639	2,006,592

Notes: Data from US Census Bureau Household Pulse Survey, Waves 1–20, 22–33.

**Table 5:** Odds ratios and 95% confidence intervals of the association of feeling hopeless to delaying medical care in previous four weeks using data from HPS waves 1–20 and 22–33 with and without demographic, health insurance, and state/time control variables.

	Delay Medical Care					
	(I) aOR (95% CI)	(II) aOR (95% CI)	(III) aOR (95% CI)	(IV) aOR (95% CI)	(V) aOR (95% CI)	(VI) aOR (95% CI)
Intercept	0.33 (0.33, 0.33)	0.42 (0.42, 0.42)	0.29 (0.29, 0.29)	0.21 (0.21, 0.22)	0.33 (0.32, 0.35)	0.15 (0.14, 0.15)
HOPELESS						
Nearly every day	4.04 (4.00, 4.08)	–	3.46 (3.42, 3.49)	4.25 (4.21, 4.30)	–	3.85 (3.81, 3.90)
More than half the days	3.12 (3.09, 3.15)	–	2.77 (2.74, 2.79)	3.11 (3.07, 3.14)	–	2.88 (2.85, 2.92)
Several days	2.20 (2.19, 2.21)	–	2.05 (2.03, 2.06)	2.13 (2.11, 2.14)	–	2.03 (2.01, 2.04)
INCOME LOSS						
Experienced	–	1.39 (1.38, 1.40)	1.24 (1.23, 1.25)	–	1.30 (1.29, 1.31)	1.22 (1.21, 1.23)
Experienced-No Response	–	1.05 (0.97, 1.13)	1.03 (0.95, 1.11)	–	1.15 (1.05, 1.25)	1.13 (1.04, 1.23)
Expected	–	1.76 (1.75, 1.78)	1.56 (1.54, 1.57)	–	1.66 (1.65, 1.67)	1.47 (1.46, 1.49)
Expected-No Response	–	1.26 (1.19, 1.34)	1.20 (1.13, 1.27)	–	1.30 (1.22, 1.39)	1.23 (1.15, 1.32)
Demographic and Spatiotemporal controls	No	No	No	Yes	Yes	Yes
Akaike Information Criterion	2,819,271	2,887,003	2,787,169	2,286,482	2,350,493	2,268,084

Notes: Data from US Census Bureau Household Pulse Survey, Waves 1–20, 22–33.

**Table 6:** Odds ratios and 95% confidence intervals of the association of feeling hopeless to foregoing or delaying medical care in previous four weeks using data from HPS waves 1–27 (Experienced Income Loss = since beginning of COVID) and 28–33 (Experienced Income Loss = last four weeks).

	Forego Medical Care			Delay Medical Care		
	(I) All Waves aOR (95% CI)	(II) Waves 1–27 aOR (95% CI)	(III) Waves 28–33 aOR (95% CI)	(IV) All Waves aOR (95% CI)	(V) Waves 1–27 aOR (95% CI)	(VI) Waves 28–33 aOR (95% CI)
Intercept	0.18 (0.18, 0.18)	0.21 (0.21, 0.21)	0.07 (0.07, 0.07)	0.29 (0.29, 0.29)	0.35 (0.35, 0.35)	0.10 (0.10, 0.10)
HOPELESS						
Nearly every day	3.93 (3.89, 3.97)	3.79 (3.75, 3.83)	5.83 (5.65, 6.03)	3.46 (3.42, 3.49)	3.36 (3.33, 3.40)	5.06 (4.91, 5.22)
More than half the days	2.92 (2.89, 2.95)	2.80 (2.77, 2.83)	4.02 (3.89, 4.17)	2.77 (2.74, 2.79)	2.66 (2.63, 2.69)	3.83 (3.71, 3.95)
Several days	2.00 (1.99, 2.01)	1.92 (1.91, 1.93)	2.58 (2.52, 2.65)	2.05 (2.03, 2.06)	1.97 (1.95, 1.98)	2.62 (2.56, 2.68)
INCOME LOSS						
Experienced	1.26 (1.25, 1.27)	1.14 (1.13, 1.15)	1.44 (1.39, 1.49)	1.24 (1.23, 1.25)	1.10 (1.10, 1.11)	1.36 (1.31, 1.40)
Experienced-No Response	1.08 (0.99, 1.17)	1.14 (1.03, 1.25)	0.87 (0.67, 1.13)	1.03 (0.95, 1.11)	1.04 (0.96, 1.14)	1.12 (0.90, 1.38)
Expected	1.65 (1.64, 1.67)	1.63 (1.61, 1.64)	1.86 (1.78, 1.93)	1.56 (1.54, 1.57)	1.53 (1.52, 1.54)	1.79 (1.73, 1.86)
Expected-No Response	1.37 (1.28, 1.46)	1.38 (1.61, 1.64)	1.43 (1.15, 1.77)	1.20 (1.13, 1.27)	1.21 (1.14, 1.29)	1.17 (0.96, 1.43)
Demographic and Spatiotemporal controls	No	No	No	No	No	No
Akaike Information Criterion	2,450,446	2,185,192	239,229	2,787,169	2,457,497	283,414

Notes: Data from US Census Bureau Household Pulse Survey, Waves 1–20, 22–33.

The relationship between hopelessness and foregoing medical care is robust and statistically significant, even after the inclusion of COVID stressors, demographic factors, health insurance status, and state and time fixed effects (Table 4). Feeling hopeless at any point in time more than doubles the likelihood of an individual foregoing needed medical care. Individuals who felt hopeless nearly every day were over 3.9 times more likely to forego needed medical care, even with the inclusion of all control variables, while experiencing hopelessness any amount of the time doubled the likelihood that an individual missed needed medical care. Income loss variables also consistently predict a greater likelihood of foregoing medical care. The estimated odds ratios are smaller for income loss variables, however, ranging from 1.25 to 1.42 times more likely to forego care for experienced income loss to 1.5 to 1.9 times more likely for expected income loss. Full regression results, including demographic variables, health insurance, and fixed effects, are reported in S4 Table. Interestingly, while we found that individuals who were older, more educated, and had health insurance were less likely to feel hopeless, these variables predict a greater likelihood of foregoing medical care. Being female is also related to a greater likelihood of foregoing medical care, while higher income is associated with a decreased likelihood of foregone medical care.

Findings are similar for the relationship between hopelessness and delaying medical care (Table 5). The odds of delaying medical care are 2 times higher for any reported level of hopelessness and approximately 3.9 times higher for individuals feeling hopeless nearly every day when income loss and all control variables are included in the analysis (Table 5, column VI). Experienced income loss is associated with an increased likelihood of delaying care; in the full model, the likelihood of delaying care is 1.25 higher for an individual who experienced income loss relative to someone who has not. Individuals who expect an income loss are approximately 1.5 times more likely to delay care. The complete regression results for delayed care, including demographic variables, health insurance, and fixed effects, are reported in S5 Table. Female respondents, respondents with higher levels of education, and respondents with insurance are more likely to delay care, while respondents with higher levels of household income are less likely to delay care.

Table 6 presents comparative results for the full sample, waves 1–27, and waves 28–33 to examine the change in the definition of the experienced income loss variable from any loss experienced since the beginning of the pandemic (waves 1–27) to income loss experienced in the preceding four weeks (waves 28–33) for the forego and delay outcomes. While we examined waves 1–27 and 28–33 separately to examine changes in the estimated relationship between experienced income loss and foregoing/delaying care following the change in the temporal definition of the experienced income loss variable, we find an increase in estimated coefficients across hopelessness and income loss variables, with the largest changes occurring for hopelessness variables. For instance, the estimated odds of foregoing care for an individual feeling hopeless nearly every day increased from 3.8 in waves 1–27 to over 5.8 in waves 28–33 (see Table 6, columns II and III). On the other hand, the estimated odds of foregoing care for someone experiencing income loss increased from 1.14 in waves 1–27 to 1.44 in waves 28–33; at the same time, the odds of foregoing care for someone expecting income loss increased from 1.63 in waves 1–27 to 1.86 in waves 28–33. Similar increases in estimated relationships occur for delaying care (Table 6, columns V and VI). While it is unclear what led to these stronger estimated relationships in waves 28–33, it may be that as policy-based restrictions on access to medical care were removed, healthcare access reflected the individuals’ decisions—including affective influences on decision-making—to a greater extent than in the early survey waves.

Full results with demographic characteristics include that female respondents, individuals with higher levels of education, and those with health insurance were more likely to report foregoing or delaying healthcare (a result that has been found in some [30], though not all [53], studies, and that may reflect that individuals without health insurance would not have planned to access healthcare [54]). On the other hand, individuals with higher levels of income were less likely to forego or delay healthcare.

Table 7 presents average marginal effects of hopelessness and income loss variables on foregoing and delaying medical care outcomes for waves 1–27 and 28–33. Estimated average marginal effects show large impacts of these variables on foregoing and delaying medical care outcomes. Individuals who reported experiencing hopelessness nearly every day in the previous week had higher probability of foregoing (between 24.1 and 29.6 percentage points) and delaying (26 to 29.6 percentage points) medical care relative to individuals who experienced no days of hopelessness. Experiencing hopelessness several days or more than half the days also led to significantly higher probabilities of foregoing or delaying medical care. Hopelessness more than half the days had a 16.7 to 22.0 percentage point increase in foregoing medical care and 19.9 to 23.1 percentage point increase in delaying medical care, while the probability of foregoing (delaying) medical care with several days of hopelessness was 9.7 to 12.7 (12.6 to 15.2) percentage points higher than no hopelessness. Experiencing or expecting income loss also increased the probability of foregoing or delaying medical care. Experiencing income loss was associated with a 2.5 to 4.1 percentage point increase in foregoing medical care, while expecting income loss was associated with a 3.9 to 6.4 percentage point increase in foregoing medical care. Estimated average marginal effects for delaying medical care were similar. The increase in the probability of delaying medical care in response to experienced income loss ranged from 2.2 to 4.2 percentage points, while for expected income loss, the range was 8.1 to 9.7 percentage points higher.

**Table 7:** Average marginal effects of hopelessness and income loss variables on foregoing or delaying medical care in waves 1–27 and 28–33.

	Forego Medical Care				Delay Medical Care			
	Waves 1–27 AME (95% CI)		Waves 28–33 AME (95% CI)		Waves 1–27 AME (95% CI)		Waves 28–33 AME (95% CI)	
HOPELESS								
Nearly every day	0.294^***^ (0.001)	0.298^***^ (0.001)	0.241^***^ (0.003)	0.258^***^ (0.003)	0.281^***^ (0.001)	0.296^***^ (0.001)	0.260^***^ (0.003)	0.270^***^ (0.003)
More than half the days	0.220^***^ (0.001)	0.219^***^ (0.001)	0.167^***^ (0.003)	0.185^***^ (0.003)	0.226^***^ (0.001)	0.231^***^ (0.001)	0.199^***^ (0.003)	0.209^***^ (0.003)
Several days	0.127^***^ (0.001)	0.125^***^ (0.001)	0.097^***^ (0.001)	0.103^***^ (0.002)	0.152^***^ (0.001)	0.149^***^ (0.001)	0.126^***^ (0.002)	0.127^***^ (0.002)
INCOME LOSS								
Experienced	0.025^***^ (0.001)	0.040^***^ (0.001)	0.041^***^ (0.002)	0.038^***^ (0.002)	0.022^***^ (0.001)	0.041^***^ (0.001)	0.042^***^ (0.002)	0.042^***^ (0.002)
Experienced-No Response	0.026^**^ (0.001)	0.030^**^ (0.010)	-0.014 (0.012)	-0.016 (0.012)	0.009 (0.010)	0.025^*^ (0.011)	0.015 (0.015)	0.024 (0.016)
Expected	0.098^***^ (0.001)	0.076^***^ (0.001)	0.076^***^ (0.003)	0.068^***^ (0.003)	0.097^***^ (0.001)	0.082^***^ (0.001)	0.087^***^ (0.003)	0.081^***^ (0.003)
Expected-No Response	0.064^***^ (0.007)	0.053^***^ (0.008)	0.040^**^ (0.014)	0.039^**^ (0.014)	0.043^***^ (0.007)	0.046^***^ (0.008)	0.021 (0.014)	0.023 (0.014)
Demographic and Spatiotemporal controls	No	Yes	No	Yes	No	Yes	No	Yes

### 3.3 Mediation analysis

**Fig 6:**
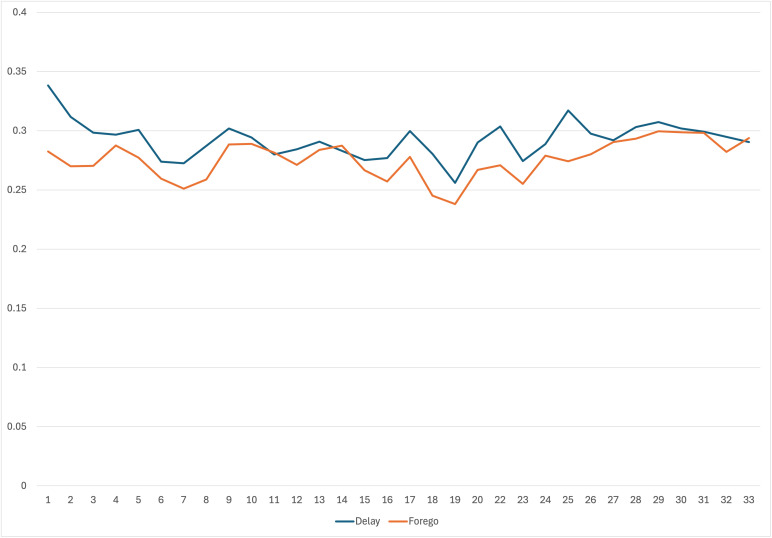
The proportion of the relationship between COVID-related income loss and delaying or foregoing healthcare mediated by hopelessness in analyses including all control variables.

Mediation analysis identifies that hopelessness is a significant mediator of the relationship between the income loss-related COVID-19 stressors and foregoing or delaying needed medical care (Fig 6). Analyses used week-specific mediation, as the full sample was too large for available computing resources to process. The mediation analyses examine the direct and indirect impacts of income loss on foregoing and delaying medical care. Results revealed that hopelessness mediated 32–40 percent of the relationship between income loss and foregoing needed medical care and 33–51 percent of the relationship between income loss and delaying medical care when demographic control variables, health insurance, and state-level fixed effects were not included. When these control variables were included, the mediated effect of hopelessness on foregoing needed medical care ranged between 25–30 percent for foregoing needed medical care and 26–34 percent on delaying medical care. While there are clear temporal trends in income loss, hopelessness, and foregone/delayed healthcare in the raw data, the estimated role of hopelessness in mediating the relationship between income loss and foregone/delayed care does not reflect these changes, suggesting that hopelessness plays a stable role in explaining the relationship between income loss and foregone/delayed healthcare access.

## 4. Discussion

Strong affective responses can be generated by societal-level events, such as disease outbreaks and pandemics [55,56], natural disasters linked to climate change [57], unrest and violence [58,59], and political polarization [60,61]. While these events can directly affect behavior by, for instance, yielding legislation delaying non-critical surgical procedures or by making public spaces a potential route for disease transmission, our findings suggest another potentially far-reaching impact: these events may lead to behavioral changes that are not directly related to the event itself, but that are instead the result of affect-mediated impacts of the event on subsequent decision-making. Individuals may become more averse to negative findings in their choices about seeking medical care, want to avoid learning about potential health concerns due to a heightened sense of fear and uncertainty, or have affect-mediated behavioral differences from those who experience less of an emotional response [56,59,62,63].

This study provides consistent, robust evidence of a relationship among exposure to income loss resulting from the COVID-19 pandemic, hopelessness, and foregoing or delaying healthcare. Specifically, the estimated mediation of the relationship between COVID-19-related income loss and delayed/foregone healthcare by the mediator variable (hopelessness) is highly consistent throughout the study period, despite significant variation in the pandemic conditions and while controlling for differences in state-level policies and demographic variables. Marital status and household size were not included in the set of demographic variables used as controls in the analysis in this study. A deciding factor for not including these variables is that the relationship between relationship status and mental health outcomes depends on the quality of the relationship. This link between relationship status and mental health has been found to hold in numerous studies [64]; the pattern also appeared to hold during the pandemic: individuals in “bad” relationships had worse outcomes than individuals in no relationship, while individuals in “good” relationships had better outcomes than those without partners [65,66]. Relationship quality also likely has an important interactive effect with the children in the household (which may explain why research on the impact of early pandemic public health measures restricting movement in the US found that women’s mental health outcomes were not explained significantly by children [67]. However, information on relationship quality was not collected in the HPS surveys, leading us to omit those.

While the sheer number of responses offered by the HPS is a strength of this research, a primary limitation is that the cross-sectional nature of the waves of survey data does not permit causal interpretations of the estimates. However, previous literature and some of our results may hint at directionality of the relationships. We will next discuss evidence for exogeneity of income loss, impact of income loss on affect, and relationships between hopelessness and healthcare access.

Previous literature documents increases in unemployment claims during the early phases of the pandemic [68], which indicates a loss of income that is exogenous to the individual [69], as unemployment can generally not be claimed by those voluntarily giving up employment. Multiple studies find that pandemic-related income loss negatively impacted mental health [37,70–74]. In fact, a longitudinal cohort study documents that income loss during the first six months of the pandemic led to long-term impacts: individuals who experienced income loss had higher levels of psychological distress two years after the income loss was experienced [72].

The relationship between hopelessness and healthcare access is complex. Hopelessness increases avoidance of tests or negative information [6–8], but being unable to access needed care can also negatively impact mental health [53]. Surveys showed that the most common reason for not getting care during 2020 was fear of COVID-19 (44%) [53]; as noted earlier, negative affective states can lead to greater perceived risks of negative events [3,4]. The second most commonly cited cause for not receiving care was that the provider cancelled the appointment (25%) [53]. In a follow-up survey round in the same study, there was a slight increase in the percentage of respondents indicating fear (47%) as the reason for not accessing care, but a drop in the provider-cancelled responses (14%), which may reflect patterns observed in our results. Specifically, evidence from the two HPS sub-samples generated by the change in definition of the experienced income loss variable provides evidence of the relationship between hopelessness and healthcare access due to changes in the prevalence of institutional or policy-driven restrictions on healthcare access during the early (waves 1–27) and late (waves 28–33) phases of the study period. During the early phase, 32 states implemented orders suspending elective medical procedures (during waves 1–27); however, these orders had all been rescinded prior to waves 28–33 [75]. The early period corresponds to markedly higher percentages of respondents reporting foregoing or delaying care in the HPS data (see Fig 1). If feelings of hopelessness primarily reflected an inability to access healthcare due to policies suspending certain types of care, then the strength of the estimated relationship between hopelessness and foregone/delayed care should not have changed between waves 1–27–28–33. Instead, we see markedly stronger relationships between hopelessness and foregoing/delaying healthcare in waves 28–33 than in waves 1–27, indicating that differences in feelings of hopelessness had a stronger relationship with healthcare access outcomes after restrictions had been removed. While previous literature suggests a bidirectional relationship between hopelessness and healthcare access, the strengthening of the relationship between hopelessness levels and healthcare access outcomes after the removal of exogenous policy-mandated limitations in access to healthcare suggests that feelings of hopelessness likely did drive some decisions to access healthcare.

Our findings highlight the connections among multiple factors that importantly impact people’s wellbeing during a global pandemic. While the data do not permit establishment of causality, previous research found higher levels of information avoidance in a designed experiment for people with higher levels of hopelessness linked to COVID-19-related income loss [22], which hints at secondary, affect-driven impacts of stressful societal or global events. Further research could examine the multi-faceted impacts of crises on decision-making, particularly those related to affect and mental health. Evidence suggests that the timing of government support disbursements during the COVID-19 pandemic correlated with improvements in mental health outcomes [76].

This study investigated the relationship between COVID-19-related stressors, hopelessness, and two important health outcomes: foregoing or delaying needed medical care. The findings reveal strong positive relationships between stressors, more frequent feelings of hopelessness, and not obtaining medical care. While the percentages of respondents reporting delaying or foregoing care declined markedly across the study period, the estimated role of hopelessness in mediating the relationship between income loss and delaying/foregoing care was highly stable across the study period, suggesting that hopelessness plays an important role in mediating the relationship between stressors and healthcare outcomes. A decrease in mental health due to COVID-19 has been widely noted previously, but these results underscore a broader implication: threats to mental health can influence decision-making in important areas beyond those directly related to the crisis. Future research should examine the wider ramifications of affective responses to crises on decision-making in other important domains.

## Supporting information

S1 Table Summary statistics and tests of differences in demographic variables of participants reporting and omitting data about hopelessness and/or delaying/foregoing medical care. (DOCX)

S2 Table Week-specific survey invitations, responses, and response rate. (DOCX)

S3 Table Full results of ordered logistic regression analysis of the relationship of hopelessness to income loss and all demographic, state, and time control variables. (DOCX)

S4 Table Full results of logistic regression analysis of the relationship of foregoing medical care with hopelessness, income loss, and all control variables. (XLSX)

S5 Table Full results of logistic regression analysis of the relationship of delaying medical care with hopelessness, income loss, and all control variables. (XLSX)
