# Supplementary material for: The role of hopelessness in mediating the relationship between income loss and delaying and foregoing healthcare: Evidence from repeated cross-sectional waves of the Household Pulse Survey
Source: PLOS Ment Health. 2025 Jul 31;2(7):e0000395. doi: 10.1371/journal.pmen.0000395 (PMC12798331; doi:10.1371/journal.pmen.0000395)
Supplement: S1 Table — (DOCX) [file pmen.0000395.s001.docx]

S1 Table: Summary statistics and tests of differences in demographic variables of participants reporting and omitting data about hopelessness and/or delaying/foregoing medical care.

|  | Reporting | Omitting | p-value |
| --- | --- | --- | --- |
| **Female** (%) | 59.3 | 59.8 | <0.001 |
| **Age** (years) | 52.6 | 49.8 | <0.001 |
| **Education** (%) |  |  | <0.001 |
| Less than high school | 0.5 | 1.2 |  |
| Some high school | 1.2 | 2.7 |  |
| High school/GED | 10.9 | 15.9 |  |
| Some college | 21.0 | 24.5 |  |
| Associate’s degree | 10.5 | 10.8 |  |
| Bachelor’s degree | 29.5 | 25.7 |  |
| Graduate degree | 26.3 | 19.1 |  |
| **Income** (%) |  |  | <0.001 |
| <$25,000 | 9.8 | 0.3 |  |
| $25,000-34,999 | 8.2 | 0.2 |  |
| $35,000-49,999 | 10.3 | 0.2 |  |
| $50,000-74,999 | 16.6 | 0.3 |  |
| $75,000-99,999 | 13.9 | 0.2 |  |
| $100,000-149,999 | 17.3 | 0.3 |  |
| $150,0000-199,999 | 8.4 | 0.1 |  |
| ≥$200,000 | 9.9 | 0.2 |  |
| PNR | 5.7 | 98.2 |  |
| **Hispanic/Latino** (%) | 8.5 | 12.2 | <0.001 |
| **Race** (%) |  |  | <0.001 |
| White alone | 83.3 | 77.1 |  |
| Black alone | 7.3 | 11.4 |  |
| Asian alone | 4.7 | 6.0 |  |
| Other/Multiple | 4.7 | 5.5 |  |

Notes: Distributions were tested using Chi-square tests for Female, Education, Income, Hispanic/Latino (all categorical variables), and Race and t-test for Age (continuous).
