# Supplementary material for: The role of hopelessness in mediating the relationship between income loss and delaying and foregoing healthcare: Evidence from repeated cross-sectional waves of the Household Pulse Survey
Source: PLOS Ment Health. 2025 Jul 31;2(7):e0000395. doi: 10.1371/journal.pmen.0000395 (PMC12798331; doi:10.1371/journal.pmen.0000395)
Supplement: S2 Table — (DOCX) [file pmen.0000395.s002.docx]

S2 Table: Week-specific survey invitations, responses, and response rate

| Week | Number of Survey Invitations | Responses | Response Rate (%) |
| --- | --- | --- | --- |
| 1 | 1,867,126 | 74,413 | 4.0 |
| 2 | 1,046,941 | 41,996 | 4.0 |
| 3 | 1,286,674 | 132,961 | 10.3 |
| 4 | 1,159,411 | 101,215 | 8.7 |
| 5 | 1,070,820 | 105,066 | 9.8 |
| 6 | 979,236 | 83,302 | 8.5 |
| 7 | 1,172,900 | 73,472 | 6.3 |
| 8 | 1,309,591 | 108,062 | 8.3 |
| 9 | 1,061,047 | 98,663 | 9.3 |
| 10 | 1,016,127 | 90,767 | 8.9 |
| 11 | 1,095,187 | 91,605 | 8.4 |
| 12 | 1,122,702 | 86,792 | 7.7 |
| 13 | 1,032,959 | 109,051 | 10.6 |
| 14 | 1,033,494 | 110,019 | 10.6 |
| 15 | 1,034,047 | 99,302 | 9.6 |
| 16 | 1,034,605 | 95,604 | 9.2 |
| 17 | 1,035,186 | 88,716 | 8.6 |
| 18 | 1,035,752 | 58,729 | 5.7 |
| 19 | 1,036,354 | 71,939 | 6.9 |
| 20 | 1,036,968 | 72,484 | 7.0 |
| 22 | 1,037,972 | 68,348 | 6.6 |
| 23 | 1,038,530 | 80,567 | 7.8 |
| 24 | 1,038,804 | 77,122 | 7.4 |
| 25 | 1,039,370 | 77,788 | 7.5 |
| 26 | 1,039,796 | 78,306 | 7.5 |
| 27 | 1,040,111 | 77,104 | 7.4 |
| 28 | 1,040,387 | 68,913 | 6.6 |
| 29 | 1,040,864 | 78,467 | 7.5 |
| 30 | 1,041,261 | 72,897 | 7.0 |
| 31 | 1,041,572 | 70,854 | 6.8 |
| 32 | 1,041,827 | 68,067 | 6.5 |
| 33 | 1,042,285 | 66,262 | 6.4 |
| Total | 34,949,906 | 2,678,853 | 7.7 |

Source: <https://www.census.gov/programs-surveys/household-pulse-survey/technical-documentation/source-accuracy.html>
