# Supplementary material for: The role of hopelessness in mediating the relationship between income loss and delaying and foregoing healthcare: Evidence from repeated cross-sectional waves of the Household Pulse Survey
Source: PLOS Ment Health. 2025 Jul 31;2(7):e0000395. doi: 10.1371/journal.pmen.0000395 (PMC12798331; doi:10.1371/journal.pmen.0000395)
Supplement: S3 Table — (DOCX) [file pmen.0000395.s003.docx]

I. Full results of ordered logistic regression analysis of the relationship of hopelessness to income loss and all demographic, state, and time control variables (Waves 1-33)

|  | **as.factor(HOPELESS)** | | |
| --- | --- | --- | --- |
| *Predictors* | *Odds Ratios* | *CI* | *p* |
| 1\|2 | 0.29 | 0.28 – 0.30 | **<0.001** |
| 2\|3 | 1.44 | 1.40 – 1.49 | **<0.001** |
| 3\|4 | 3.35 | 3.24 – 3.46 | **<0.001** |
| INCOMELOSS EXPERIENCED [1] | 1.40 | 1.39 – 1.41 | **<0.001** |
| INCOMELOSS EXPERIENCED [PNR] | 1.09 | 1.01 – 1.18 | **0.024** |
| INCOMELOSS EXPECTED [1] | 1.72 | 1.71 – 1.73 | **<0.001** |
| INCOMELOSS EXPECTED [PNR] | 1.34 | 1.27 – 1.43 | **<0.001** |
| FEMALE | 1.30 | 1.30 – 1.31 | **<0.001** |
| AGE | 0.98 | 0.98 – 0.98 | **<0.001** |
| EDUCATION | 0.99 | 0.99 – 0.99 | **<0.001** |
| INCOME [2] | 0.76 | 0.75 – 0.77 | **<0.001** |
| INCOME [3] | 0.66 | 0.66 – 0.67 | **<0.001** |
| INCOME [4] | 0.56 | 0.56 – 0.57 | **<0.001** |
| INCOME [5] | 0.48 | 0.47 – 0.48 | **<0.001** |
| INCOME [6] | 0.41 | 0.41 – 0.42 | **<0.001** |
| INCOME [7] | 0.37 | 0.36 – 0.37 | **<0.001** |
| INCOME [8] | 0.32 | 0.31 – 0.32 | **<0.001** |
| INCOME [PNR] | 0.42 | 0.42 – 0.43 | **<0.001** |
| INSURANCE | 0.80 | 0.79 – 0.81 | **<0.001** |
| WEEK [2] | 1.06 | 1.04 – 1.09 | **<0.001** |
| WEEK [3] | 1.10 | 1.07 – 1.12 | **<0.001** |
| WEEK [4] | 1.11 | 1.08 – 1.13 | **<0.001** |
| WEEK [5] | 1.14 | 1.11 – 1.16 | **<0.001** |
| WEEK [6] | 1.15 | 1.13 – 1.17 | **<0.001** |
| WEEK [7] | 1.19 | 1.16 – 1.21 | **<0.001** |
| WEEK [8] | 1.16 | 1.14 – 1.19 | **<0.001** |
| WEEK [9] | 1.23 | 1.21 – 1.26 | **<0.001** |
| WEEK [10] | 1.26 | 1.23 – 1.28 | **<0.001** |
| WEEK [11] | 1.30 | 1.27 – 1.32 | **<0.001** |
| WEEK [12] | 1.31 | 1.28 – 1.34 | **<0.001** |
| WEEK [13] | 1.22 | 1.19 – 1.24 | **<0.001** |
| WEEK [14] | 1.21 | 1.18 – 1.24 | **<0.001** |
| WEEK [15] | 1.29 | 1.27 – 1.32 | **<0.001** |
| WEEK [16] | 1.26 | 1.23 – 1.29 | **<0.001** |
| WEEK [17] | 1.30 | 1.27 – 1.32 | **<0.001** |
| WEEK [18] | 1.42 | 1.39 – 1.46 | **<0.001** |
| WEEK [19] | 1.46 | 1.43 – 1.49 | **<0.001** |
| WEEK [20] | 1.42 | 1.39 – 1.45 | **<0.001** |
| WEEK [22] | 1.51 | 1.48 – 1.54 | **<0.001** |
| WEEK [23] | 1.42 | 1.39 – 1.45 | **<0.001** |
| WEEK [24] | 1.40 | 1.37 – 1.43 | **<0.001** |
| WEEK [25] | 1.35 | 1.32 – 1.38 | **<0.001** |
| WEEK [26] | 1.25 | 1.23 – 1.28 | **<0.001** |
| WEEK [27] | 1.18 | 1.16 – 1.21 | **<0.001** |
| WEEK [28] | 1.11 | 1.09 – 1.14 | **<0.001** |
| WEEK [29] | 1.05 | 1.03 – 1.07 | **<0.001** |
| WEEK [30] | 1.04 | 1.02 – 1.06 | **0.001** |
| WEEK [31] | 0.98 | 0.96 – 1.01 | 0.141 |
| WEEK [32] | 1.00 | 0.97 – 1.02 | 0.668 |
| WEEK [33] | 0.96 | 0.94 – 0.98 | **<0.001** |
| EST ST [2] | 0.98 | 0.95 – 1.01 | 0.168 |
| EST ST [4] | 1.07 | 1.04 – 1.10 | **<0.001** |
| EST ST [5] | 1.07 | 1.03 – 1.11 | **<0.001** |
| EST ST [6] | 1.25 | 1.22 – 1.28 | **<0.001** |
| EST ST [8] | 1.09 | 1.05 – 1.12 | **<0.001** |
| EST ST [9] | 1.10 | 1.07 – 1.14 | **<0.001** |
| EST ST [10] | 0.96 | 0.93 – 1.00 | **0.030** |
| EST ST [11] | 1.34 | 1.30 – 1.39 | **<0.001** |
| EST ST [12] | 1.04 | 1.01 – 1.07 | **0.008** |
| EST ST [13] | 0.98 | 0.96 – 1.01 | 0.305 |
| EST ST [15] | 0.93 | 0.90 – 0.97 | **<0.001** |
| EST ST [16] | 0.92 | 0.89 – 0.95 | **<0.001** |
| EST ST [17] | 1.10 | 1.06 – 1.13 | **<0.001** |
| EST ST [18] | 0.95 | 0.92 – 0.98 | **0.002** |
| EST ST [19] | 0.93 | 0.90 – 0.96 | **<0.001** |
| EST ST [20] | 0.97 | 0.94 – 1.00 | 0.088 |
| EST ST [21] | 1.08 | 1.05 – 1.12 | **<0.001** |
| EST ST [22] | 1.10 | 1.06 – 1.13 | **<0.001** |
| EST ST [23] | 1.00 | 0.96 – 1.04 | 0.967 |
| EST ST [24] | 1.14 | 1.11 – 1.18 | **<0.001** |
| EST ST [25] | 1.09 | 1.06 – 1.12 | **<0.001** |
| EST ST [26] | 0.96 | 0.94 – 0.99 | **0.014** |
| EST ST [27] | 0.89 | 0.86 – 0.92 | **<0.001** |
| EST ST [28] | 1.01 | 0.97 – 1.05 | 0.647 |
| EST ST [29] | 0.98 | 0.95 – 1.02 | 0.316 |
| EST ST [30] | 0.86 | 0.83 – 0.89 | **<0.001** |
| EST ST [31] | 0.91 | 0.88 – 0.94 | **<0.001** |
| EST ST [32] | 1.08 | 1.05 – 1.12 | **<0.001** |
| EST ST [33] | 1.01 | 0.98 – 1.05 | 0.488 |
| EST ST [34] | 1.17 | 1.13 – 1.21 | **<0.001** |
| EST ST [35] | 1.15 | 1.11 – 1.19 | **<0.001** |
| EST ST [36] | 1.15 | 1.12 – 1.19 | **<0.001** |
| EST ST [37] | 0.96 | 0.93 – 0.99 | **0.015** |
| EST ST [38] | 0.80 | 0.77 – 0.83 | **<0.001** |
| EST ST [39] | 1.00 | 0.97 – 1.04 | 0.781 |
| EST ST [40] | 1.07 | 1.04 – 1.11 | **<0.001** |
| EST ST [41] | 1.22 | 1.18 – 1.26 | **<0.001** |
| EST ST [42] | 1.09 | 1.06 – 1.13 | **<0.001** |
| EST ST [44] | 0.98 | 0.95 – 1.02 | 0.368 |
| EST ST [45] | 0.88 | 0.85 – 0.91 | **<0.001** |
| EST ST [46] | 0.81 | 0.78 – 0.84 | **<0.001** |
| EST ST [47] | 0.97 | 0.94 – 1.00 | **0.048** |
| EST ST [48] | 1.04 | 1.01 – 1.07 | **0.006** |
| EST ST [49] | 1.01 | 0.98 – 1.04 | 0.406 |
| EST ST [50] | 1.11 | 1.08 – 1.15 | **<0.001** |
| EST ST [51] | 1.07 | 1.04 – 1.10 | **<0.001** |
| EST ST [53] | 1.20 | 1.17 – 1.23 | **<0.001** |
| EST ST [54] | 1.18 | 1.14 – 1.22 | **<0.001** |
| EST ST [55] | 0.92 | 0.89 – 0.95 | **<0.001** |
| EST ST [56] | 0.96 | 0.93 – 1.00 | 0.051 |
| Observations | 1951265 | | |
| R^2^ Nagelkerke | 0.426 | | |

II. Full results of ordered logistic regression analysis of the relationship of hopelessness to income loss and all demographic, state, and time control variables (WAVES 1-27)

|  | **as.factor(HOPELESS_DEPVAR)** | | |
| --- | --- | --- | --- |
| *Predictors* | *Odds Ratios* | *CI* | *p* |
| 1\|2 | 0.31 | 0.30 – 0.32 | **<0.001** |
| 2\|3 | 1.59 | 1.53 – 1.65 | **<0.001** |
| 3\|4 | 3.72 | 3.59 – 3.86 | **<0.001** |
| INCOMELOSS EXPERIENCED [1] | 1.37 | 1.36 – 1.38 | **<0.001** |
| INCOMELOSS EXPERIENCED [PNR] | 1.10 | 1.01 – 1.20 | **0.029** |
| INCOMELOSS EXPECTED [1] | 1.71 | 1.70 – 1.72 | **<0.001** |
| INCOMELOSS EXPECTED [PNR] | 1.27 | 1.20 – 1.36 | **<0.001** |
| FEMALE | 1.31 | 1.30 – 1.32 | **<0.001** |
| AGE | 0.98 | 0.98 – 0.98 | **<0.001** |
| EDUCATION | 0.99 | 0.99 – 0.99 | **<0.001** |
| INCOME [2] | 0.76 | 0.75 – 0.77 | **<0.001** |
| INCOME [3] | 0.67 | 0.66 – 0.68 | **<0.001** |
| INCOME [4] | 0.57 | 0.56 – 0.58 | **<0.001** |
| INCOME [5] | 0.48 | 0.48 – 0.49 | **<0.001** |
| INCOME [6] | 0.42 | 0.42 – 0.43 | **<0.001** |
| INCOME [7] | 0.38 | 0.37 – 0.38 | **<0.001** |
| INCOME [8] | 0.33 | 0.32 – 0.33 | **<0.001** |
| INCOME [PNR] | 0.43 | 0.42 – 0.44 | **<0.001** |
| INSURANCE | 0.79 | 0.78 – 0.80 | **<0.001** |
| WEEK [2] | 1.07 | 1.04 – 1.10 | **<0.001** |
| WEEK [3] | 1.09 | 1.07 – 1.12 | **<0.001** |
| WEEK [4] | 1.08 | 1.06 – 1.11 | **<0.001** |
| WEEK [5] | 1.13 | 1.11 – 1.16 | **<0.001** |
| WEEK [6] | 1.15 | 1.13 – 1.18 | **<0.001** |
| WEEK [7] | 1.18 | 1.16 – 1.21 | **<0.001** |
| WEEK [8] | 1.16 | 1.14 – 1.19 | **<0.001** |
| WEEK [9] | 1.23 | 1.21 – 1.26 | **<0.001** |
| WEEK [10] | 1.25 | 1.23 – 1.28 | **<0.001** |
| WEEK [11] | 1.28 | 1.26 – 1.31 | **<0.001** |
| WEEK [12] | 1.32 | 1.29 – 1.34 | **<0.001** |
| WEEK [13] | 1.21 | 1.18 – 1.23 | **<0.001** |
| WEEK [14] | 1.20 | 1.17 – 1.22 | **<0.001** |
| WEEK [15] | 1.28 | 1.26 – 1.31 | **<0.001** |
| WEEK [16] | 1.26 | 1.23 – 1.29 | **<0.001** |
| WEEK [17] | 1.31 | 1.28 – 1.34 | **<0.001** |
| WEEK [18] | 1.42 | 1.39 – 1.45 | **<0.001** |
| WEEK [19] | 1.46 | 1.43 – 1.50 | **<0.001** |
| WEEK [20] | 1.43 | 1.40 – 1.46 | **<0.001** |
| WEEK [22] | 1.46 | 1.42 – 1.49 | **<0.001** |
| WEEK [23] | 1.41 | 1.38 – 1.44 | **<0.001** |
| WEEK [24] | 1.40 | 1.37 – 1.43 | **<0.001** |
| WEEK [25] | 1.34 | 1.31 – 1.36 | **<0.001** |
| WEEK [26] | 1.26 | 1.24 – 1.29 | **<0.001** |
| WEEK [27] | 1.16 | 1.13 – 1.18 | **<0.001** |
| EST ST [2] | 1.01 | 0.98 – 1.04 | 0.614 |
| EST ST [4] | 1.12 | 1.08 – 1.15 | **<0.001** |
| EST ST [5] | 1.06 | 1.02 – 1.09 | **0.004** |
| EST ST [6] | 1.27 | 1.24 – 1.31 | **<0.001** |
| EST ST [8] | 1.11 | 1.08 – 1.15 | **<0.001** |
| EST ST [9] | 1.11 | 1.07 – 1.15 | **<0.001** |
| EST ST [10] | 1.06 | 1.02 – 1.10 | **0.003** |
| EST ST [11] | 1.35 | 1.30 – 1.40 | **<0.001** |
| EST ST [12] | 1.07 | 1.04 – 1.10 | **<0.001** |
| EST ST [13] | 0.99 | 0.95 – 1.02 | 0.414 |
| EST ST [15] | 0.95 | 0.92 – 0.99 | **0.009** |
| EST ST [16] | 0.93 | 0.90 – 0.96 | **<0.001** |
| EST ST [17] | 1.14 | 1.11 – 1.18 | **<0.001** |
| EST ST [18] | 1.01 | 0.98 – 1.05 | 0.540 |
| EST ST [19] | 0.91 | 0.88 – 0.94 | **<0.001** |
| EST ST [20] | 0.98 | 0.94 – 1.01 | 0.177 |
| EST ST [21] | 1.09 | 1.05 – 1.13 | **<0.001** |
| EST ST [22] | 1.11 | 1.07 – 1.15 | **<0.001** |
| EST ST [23] | 0.99 | 0.95 – 1.03 | 0.609 |
| EST ST [24] | 1.14 | 1.10 – 1.17 | **<0.001** |
| EST ST [25] | 1.11 | 1.08 – 1.15 | **<0.001** |
| EST ST [26] | 0.98 | 0.95 – 1.01 | 0.262 |
| EST ST [27] | 0.91 | 0.88 – 0.94 | **<0.001** |
| EST ST [28] | 0.98 | 0.95 – 1.02 | 0.407 |
| EST ST [29] | 1.01 | 0.98 – 1.05 | 0.528 |
| EST ST [30] | 0.88 | 0.85 – 0.91 | **<0.001** |
| EST ST [31] | 0.91 | 0.88 – 0.94 | **<0.001** |
| EST ST [32] | 1.12 | 1.08 – 1.16 | **<0.001** |
| EST ST [33] | 1.02 | 0.99 – 1.06 | 0.209 |
| EST ST [34] | 1.14 | 1.11 – 1.18 | **<0.001** |
| EST ST [35] | 1.22 | 1.17 – 1.26 | **<0.001** |
| EST ST [36] | 1.16 | 1.12 – 1.20 | **<0.001** |
| EST ST [37] | 0.97 | 0.94 – 1.01 | 0.109 |
| EST ST [38] | 0.82 | 0.79 – 0.85 | **<0.001** |
| EST ST [39] | 1.02 | 0.98 – 1.05 | 0.332 |
| EST ST [40] | 1.06 | 1.03 – 1.10 | **0.001** |
| EST ST [41] | 1.26 | 1.22 – 1.30 | **<0.001** |
| EST ST [42] | 1.14 | 1.11 – 1.18 | **<0.001** |
| EST ST [44] | 0.99 | 0.95 – 1.02 | 0.460 |
| EST ST [45] | 0.94 | 0.90 – 0.97 | **<0.001** |
| EST ST [46] | 0.81 | 0.78 – 0.84 | **<0.001** |
| EST ST [47] | 1.00 | 0.96 – 1.03 | 0.839 |
| EST ST [48] | 1.06 | 1.03 – 1.09 | **<0.001** |
| EST ST [49] | 1.02 | 0.98 – 1.05 | 0.330 |
| EST ST [50] | 1.05 | 1.01 – 1.09 | **0.008** |
| EST ST [51] | 1.10 | 1.07 – 1.14 | **<0.001** |
| EST ST [53] | 1.24 | 1.21 – 1.28 | **<0.001** |
| EST ST [54] | 1.17 | 1.13 – 1.21 | **<0.001** |
| EST ST [55] | 0.96 | 0.92 – 0.99 | **0.011** |
| EST ST [56] | 0.95 | 0.91 – 0.98 | **0.006** |
| Observations | 1615680 | | |
| R^2^ Nagelkerke | 0.465 | | |

III. Full results of ordered logistic regression analysis of the relationship of hopelessness to income loss and all demographic, state, and time control variables (WAVES 28-33)

|  | **as.factor(HOPELESS_DEPVAR)** | | |
| --- | --- | --- | --- |
| *Predictors* | *Odds Ratios* | *CI* | *p* |
| 1\|2 | 0.16 | 0.15 – 0.18 | **<0.001** |
| 2\|3 | 0.74 | 0.68 – 0.80 | **<0.001** |
| 3\|4 | 1.62 | 1.50 – 1.76 | **<0.001** |
| INCOMELOSS EXPERIENCED [1] | 1.76 | 1.71 – 1.80 | **<0.001** |
| INCOMELOSS EXPERIENCED [PNR] | 1.16 | 0.98 – 1.36 | 0.083 |
| INCOMELOSS EXPECTED [1] | 1.81 | 1.76 – 1.87 | **<0.001** |
| INCOMELOSS EXPECTED [PNR] | 1.20 | 1.03 – 1.40 | **0.019** |
| FEMALE | 1.29 | 1.27 – 1.31 | **<0.001** |
| AGE | 0.98 | 0.98 – 0.98 | **<0.001** |
| EDUCATION | 0.97 | 0.97 – 0.98 | **<0.001** |
| INCOME [2] | 0.76 | 0.73 – 0.78 | **<0.001** |
| INCOME [3] | 0.64 | 0.62 – 0.66 | **<0.001** |
| INCOME [4] | 0.53 | 0.52 – 0.54 | **<0.001** |
| INCOME [5] | 0.43 | 0.42 – 0.44 | **<0.001** |
| INCOME [6] | 0.36 | 0.35 – 0.37 | **<0.001** |
| INCOME [7] | 0.32 | 0.31 – 0.34 | **<0.001** |
| INCOME [8] | 0.26 | 0.25 – 0.27 | **<0.001** |
| INCOME [PNR] | 0.40 | 0.39 – 0.41 | **<0.001** |
| INSURANCE | 0.83 | 0.80 – 0.85 | **<0.001** |
| WEEK [29] | 0.95 | 0.93 – 0.98 | **<0.001** |
| WEEK [30] | 0.92 | 0.89 – 0.94 | **<0.001** |
| WEEK [31] | 0.90 | 0.88 – 0.92 | **<0.001** |
| WEEK [32] | 0.89 | 0.87 – 0.91 | **<0.001** |
| WEEK [33] | 0.87 | 0.85 – 0.89 | **<0.001** |
| EST ST [2] | 0.92 | 0.85 – 0.99 | **0.037** |
| EST ST [4] | 0.93 | 0.86 – 1.00 | **0.050** |
| EST ST [5] | 0.93 | 0.85 – 1.01 | 0.100 |
| EST ST [6] | 1.10 | 1.03 – 1.17 | **0.007** |
| EST ST [8] | 1.00 | 0.92 – 1.08 | 0.938 |
| EST ST [9] | 0.97 | 0.90 – 1.06 | 0.504 |
| EST ST [10] | 0.90 | 0.82 – 0.98 | **0.022** |
| EST ST [11] | 1.12 | 1.02 – 1.22 | **0.013** |
| EST ST [12] | 0.91 | 0.84 – 0.97 | **0.008** |
| EST ST [13] | 0.89 | 0.82 – 0.96 | **0.002** |
| EST ST [15] | 0.93 | 0.84 – 1.01 | 0.097 |
| EST ST [16] | 0.75 | 0.69 – 0.81 | **<0.001** |
| EST ST [17] | 0.96 | 0.89 – 1.04 | 0.312 |
| EST ST [18] | 0.84 | 0.78 – 0.91 | **<0.001** |
| EST ST [19] | 0.82 | 0.75 – 0.89 | **<0.001** |
| EST ST [20] | 0.86 | 0.79 – 0.93 | **<0.001** |
| EST ST [21] | 0.98 | 0.90 – 1.08 | 0.718 |
| EST ST [22] | 0.97 | 0.89 – 1.06 | 0.523 |
| EST ST [23] | 0.81 | 0.74 – 0.89 | **<0.001** |
| EST ST [24] | 1.02 | 0.94 – 1.10 | 0.684 |
| EST ST [25] | 0.98 | 0.90 – 1.05 | 0.514 |
| EST ST [26] | 0.89 | 0.83 – 0.96 | **0.003** |
| EST ST [27] | 0.81 | 0.75 – 0.88 | **<0.001** |
| EST ST [28] | 0.89 | 0.81 – 0.98 | **0.024** |
| EST ST [29] | 0.87 | 0.80 – 0.95 | **0.001** |
| EST ST [30] | 0.80 | 0.73 – 0.88 | **<0.001** |
| EST ST [31] | 0.81 | 0.74 – 0.88 | **<0.001** |
| EST ST [32] | 0.95 | 0.87 – 1.03 | 0.191 |
| EST ST [33] | 0.88 | 0.81 – 0.96 | **0.003** |
| EST ST [34] | 0.99 | 0.91 – 1.07 | 0.832 |
| EST ST [35] | 1.02 | 0.94 – 1.10 | 0.617 |
| EST ST [36] | 1.08 | 1.00 – 1.17 | **0.046** |
| EST ST [37] | 0.85 | 0.78 – 0.92 | **<0.001** |
| EST ST [38] | 0.73 | 0.66 – 0.81 | **<0.001** |
| EST ST [39] | 0.92 | 0.84 – 0.99 | **0.038** |
| EST ST [40] | 0.97 | 0.89 – 1.06 | 0.495 |
| EST ST [41] | 1.07 | 0.99 – 1.15 | 0.075 |
| EST ST [42] | 0.95 | 0.88 – 1.02 | 0.177 |
| EST ST [44] | 0.87 | 0.79 – 0.96 | **0.004** |
| EST ST [45] | 0.79 | 0.72 – 0.86 | **<0.001** |
| EST ST [46] | 0.70 | 0.64 – 0.78 | **<0.001** |
| EST ST [47] | 0.94 | 0.87 – 1.02 | 0.153 |
| EST ST [48] | 0.93 | 0.87 – 1.00 | **0.041** |
| EST ST [49] | 0.90 | 0.83 – 0.97 | **0.006** |
| EST ST [50] | 0.87 | 0.79 – 0.95 | **0.003** |
| EST ST [51] | 0.94 | 0.87 – 1.02 | 0.122 |
| EST ST [53] | 1.04 | 0.96 – 1.11 | 0.329 |
| EST ST [54] | 1.07 | 0.97 – 1.17 | 0.173 |
| EST ST [55] | 0.81 | 0.74 – 0.88 | **<0.001** |
| EST ST [56] | 0.86 | 0.78 – 0.95 | **0.004** |
| Observations | 335585 | | |
| R^2^ Nagelkerke | 0.154 | | |
